# The effect of community-based health management on the health of the elderly: a randomized controlled trial from China

**DOI:** 10.1186/1472-6963-12-449

**Published:** 2012-12-07

**Authors:** Jianqian Chao, Yimin Wang, Hui Xu, Qing Yu, Lili Jiang, Lin Tian, Wenyuan Xie, Pei Liu

**Affiliations:** 1 Department of Medical Insurance, School of Public Health, Southeast University, Nanjing, Jiangsu, China; 2 Hospital of Qinghuai, Nanjing, Jiangsu, China; 3 Department of Epidemiology and Biostatistics, School of Public Health, Southeast University, Nanjing, Jiangsu, China

**Keywords:** Community-based health management, Randomized control trial, Elderly Chinese, Effect

## Abstract

**Background:**

An aging population poses significant challenges to health care in China. Health management has been implemented to reduce the costs of care, raise health service utilization, increase health knowledge and improve quality of life. Several studies have tried to verify the effectiveness of health management in achieving these goals worldwide. However, there have been insufficient randomized control trials (RCTs) to draw reliable conclusions. The few small-scale studies conducted in China include mostly the general population rather than the elderly. Our study is designed to evaluate the impact of community-based health management on the health of the elderly through an RCT in Nanjing, China.

**Methods:**

Two thousand four hundred participants, aged 60 or older and who gave informed consent, were randomly allocated 1:1 into management and control groups, the randomization schedule was concealed from community health service center staff until allocation. Community-based health management was applied in the former while the latter was only given usual care. After 18 months, three categories of variables (subjective grading health indices, objective health indices and health service utilization) were measured based on a questionnaire, clinical monitoring and diagnostic measurements. Differences between the two groups were assessed before and after the intervention and analyzed with *t*-test, χ^2^-test, and multiple regression analysis.

**Results:**

Compared with the control group, the management group demonstrated improvement on the following variables (P<0.01): health knowledge score, self-evaluated psychological conditions, overall self-evaluated health conditions, diet score, physical activity duration per week, regular blood pressure monitoring, waist-to-hip ratio, systolic blood pressure and fasting blood sugar. The number of outpatient clinic visits did not differ significantly (P=0.60) between the two groups before intervention, while after intervention it was smaller in the management group than in the control group (P<0.01). However, the number of hospital admissions in the preceding 6 months was not different between the two groups even after intervention (P=0.36). Multiple regression analysis showed that gender, age, education level, chronic disease status and self-evaluated psychological conditions were important factors affecting health knowledge score, BMI, and waist-to-hip ratio.

**Conclusion:**

Community-based health management improved both subjective grading health indices, objective health indices and decreased the number of outpatient clinic visits, demonstrating effectiveness in improving elderly health.

**Trial registration:**

ChiCTR-OCH-11001716

## Background

As a developing country with one fourth of the total world population, China is facing the problem of a large and rapidly increasing elderly population. The sixth population census in 2010 showed that the population aged 60 and over was 177,648,705 (13.26%), and that aged 65 and over, 118,831,709 (8.87%) [1]. It is predicted that the elderly population over the age of 60 will increase from 144 million (11% in the total population) in 2005 up to over 437 million (30%) in 2051, with the number and accelerating speed of aging of the population in China ranking first on the globe. The average increase in the elderly population is 2-fold higher than in some Western developed countries [2]. The Nanjing region, the location of this trial, is one of the most developed regions in China (total population of 8,004,680 and 60+ population of 1,248,730 or 15.6%, which is higher than the national average [3]). The rapid increase in the elderly population not only greatly influences the development of the social economy, but also poses significant challenges to health care in China. Health management has been looked upon as a way to deal with these challenges [4-6].

There have been a number of studies of health management. Since 1987, the US Union Pacific Railroad Company has provided health management services for their employees. Apart from a great improvement of health indices in the population, the economic benefit has been obvious, with a benefit-cost ratio of 3.24:1, i.e., there is a benefit of $3.24 for each dollar spent on health management. Health management helped the company save about 40 million dollars per year [7]. Christian et al. indicated that the future development of health management programs has the potential to reduce the overall financial burden of global health care. The implementation of health promotion and education as key elements in health management may improve quality of life and patient satisfaction [8]. Therefore, implementation of health management for the elderly has the potential for significant impacts on the application of health resources, including decreases in medical costs and improvements in the health of the elderly. Recently, Hunter et al. reviewed the research literature on health management in Europe from July 1995 to June 2005 in terms of quality, range and shortcomings of the research, and indicated that studies on health management in Europe were rare, those specially designed for the elderly were even less common, and their results were not consistent [9]. However, with a randomized controlled intervention trial, Benabei et al. did demonstrate that integrated social and medical care with a case management program may provide a cost-effective approach to reduce admissions to institutions and to halt functional decline in the elderly living in the community [10]. With the same type of trial, Harari investigated the effect of health risk factors evaluation, one of the steps in elderly health management. He has concluded that this step only slightly improved health behavior and prevention service acceptance in the elderly, and that more trials will be required to arrive at a definite conclusion [11].

In China, research on health management has been carried out [12,13]. Adopting a random sampling method to select 120 elderly over the age of 60, Wang et al. compared health service demand and health knowledge before and after implementation of health management and showed that health management could improve the health and life quality of the elderly by raising health service utilization and health knowledge [14]. Because of the small sample size of this study as well as a lack of randomized controlled trials for the health management of the elderly in China, the effectiveness of health management has been inconclusive so far.

The aim of this study was to evaluate the effects of community-based health management on the health of the elderly by using an RCT design. We focused on the following questions: 1) To what extent is health management effective in improving health-related indices? 2) What are the main factors affecting improvement of the health of elderly Chinese? We expected that health management might improve health-related indices in the subjective grading items, the objective measured indices, and health service utilization. We also hypothesized that community-based health management might reduce outpatient care visits and hospital admissions.

## Methods

### Study design

The study was carried out as a randomized parallel controlled trial. It was conducted in collaboration with the Nanjing Community Health Service Center. Nanjing is located in southeastern China and is the provincial capital of Jiangsu province—one of the most developed provinces in China.

The participants were recruited from the community governed by this Community Health Service Center in 2009. The study had obtained the approval of the Medical Ethics Committee of Southeast University.

### Study population

The baseline investigation was performed in January 2009. To recruit sufficient participants, community health service center staff put up posters/notices and carried out oral propagation in the districts of the community health service center before the investigation began. A total of 2400 participants who signed informed consent forms were selected from the community after passing the inclusion and exclusion criteria stated below.

Criteria for the inclusion of participants were: 1) aged 60 and over; and 2) local permanent resident. The exclusion criteria [15] were: 1) cognitive defect, severe psychological disorder or mental illness; 2) severe chronic diseases such as heart failure, respiratory failure, liver cirrhosis, renal failure or need for assistance in living; 3) limitations in physical activity; and 4) participating in or having participated in other trials within the last 30 days.

**Figure 1:**
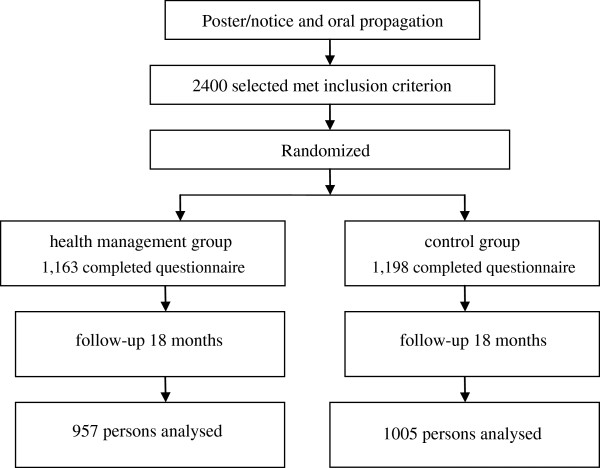
Flow diagram of participants’ progress in the randomized trial.

The selected participants were randomly allocated to either the intervention or the control group at a 1:1 ratio using a random number table. To prevent selection bias, a statistician who is not associated with the study generated a list of random numbers using a random numbers table, the randomization schedule was concealed from community health service center staff until allocation. People who lived together and in the same family were adjusted into the same group if divided into different groups to avoid the contamination effect of intervention. Flow diagram of the progress in the randomized trial is showed in Figure 1.

### Sample size consideration

Limited by the above inclusion and exclusion criteria, the sample size was determined based on the method of Stuck et al. [16]. In order to detect a postulated 30% difference in various variables in the health management group as compared with the control group at 18-month follow-up, we calculated the required sample size at a two-sided significance level of 0.05 and with a power of 80%. With 18-month follow-up and an assumed 20% drop-out rate, the sample size was determined to be a total of 2400 participants from which both the health management and control groups would be randomized in a 1:1 ratio.

### Intervention

**Figure 2:**
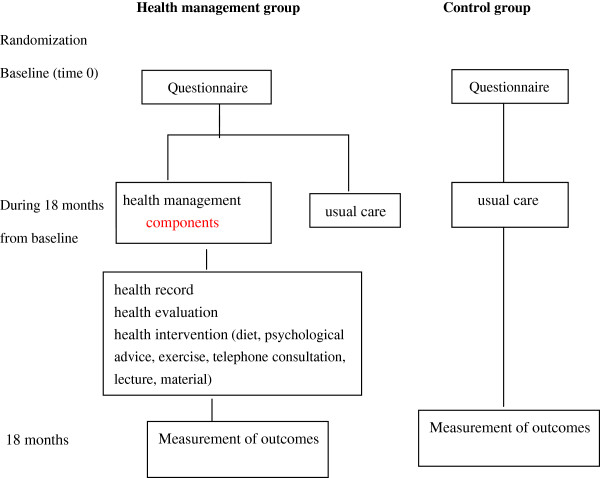
Graphical depiction of the intervention.

The intervention group received a health management program including the following components: 1) Health record establishment; 2) Health evaluation; and 3) Health management, including diet advice, psychological aspects of health, a tailor-made exercise program based on an earlier evaluation, education/skills training on health self-management, telephone consultation, lectures on health, and distribution of health promoting materials. The components of the intervention were ‘administered’ at least once per month by specifically-trained community health service center staff, managers and related researchers (Figure 2). The interventions and the compliance with the given advice were monitored. The health management group intervention lasted for 18 months. The control group received usual care. After 18 months (June 2010), measurements were performed on both the management and the control groups.

### Effect evaluation

The evaluation of the intervention effects was based on the change of health-related indices. Height and weight of the participants were measured (with a Model GRZ-120 human body weight gauge), and then BMI was calculated according the formula of weight/height^2^. Waist and hip circumferences were measured according to the WHO MONICA guidelines. Blood pressure was measured using the brachial artery in the upper right arm with participants in the sitting position. A mercury sphygmomanometer was used. Blood pressure was measured three times and the average value was used as the outcome [17]. The above indices were measured by a medically-trained community physician. Fasting blood sugar and blood triglyceride were measured by the clinical test center of the hospital. Other indices such as diet, health knowledge score and physical activity duration were obtained by specially-designed questionnaires.

A questionnaire designed by the investigators was used to measure health-related indices. Before the full-scale formal investigation, a pilot investigation was carried out in some elderly and the questionnaire was modified according to the results of the pilot investigation. Any inconsistencies, such as missing or incorrectly recorded data, occurring in the investigation were checked and re-recorded again in time to ensure the quality of the investigational data. Cronbach’s Alpha coefficient of internal consistency was 0.789, reaching the standard of 0.70, which demonstrated good reliability in the questionnaire. For face validity and content validity, relevant experts were invited to discuss the design of the questionnaire. Items inappropriately expressed were modified to ensure surface and content validity.

### Data analysis

The data were entered by a data entry operator at the first pass and then verified by an independent operator on a different computer on a second pass with Epidata 3.1 software (http://www.epidata.dk/). Any discrepancies between the first and second pass were resolved such that the data entered were a true reflection of that recorded. The data quality was also controlled by setting up logic constraining conditions and valid value ranges of relevant variables. Before and after the intervention, related indices were compared between the management and the control groups with *t*-test (for continuous measurement data), χ2-test (for categorical variables), and multiple regression analysis. All analyses were performed in SPSS13.0 (SPSS Inc, Chicago, IL, USA), with 0.05 set as the required level of significance.

## Results

### Analysis of general conditions and health indices at baseline

Among the participants who received the baseline questionnaire, there were 1,163 persons in the management group and 1,198 persons in the control group. After 18 months, among the participants who completed the second questionnaire, there were 957 persons in the management group and 1,005 persons in the control group. The management and control groups had drop-out rates at 18-month follow-up of 17.7% (206 participants) and 16.1% (193 participants), respectively. Both dropout rates were not of statistical significance (P>0.05). The main reasons for dropping out were moving, travelling, withdrawing and death.

At the start of the study (baseline), various indices under general conditions were monitored, and the differences of these indices between the management and the control groups were not statistically significant (Table 1). Furthermore, other health-related indices were taken or measured, and all but four (Physical activity duration, BMI, female BMI and diastolic blood pressure) were not statistically different (Table 2). This suggested that the two groups were balanced and comparable at the baseline level.

**Table 1:** General conditions between the management and control groups at baseline

**Variables**	**Management group(n=957)**	**Control group(n=1005)**	***t *or χ**^**2**^	***P***
	**n(%)**	**n(%)**		
Age (mean±SD, years)	69.81±6.71	69.40±7.04	1.315*	0.189
Male	459(48.0)	471(46.9)	0.236	0.627
Female	498(52.0)	534(53.1)		
Living with children	426(44.5)	418(41.6)	1.994	0.369
Husband and wife living together	467(48.8)	510(50.7)		
Living alone	64( 6.7)	77 (7.7)		
Illiteracy	99(10.3)	87( 8.7)	3.134	0.371
Primary school	183(19.1)	177(17.6)		
High school	568(59.4)	632(62.9)		
University	107(11.2)	109(10.9)		
Married	812(84.8)	839(83.5)	0.722	0.868
Divorced or Widowed	145(15.2)	166(16.5)		
Disease-free	578(60.4)	643(64.0)	2.678	0.102
Hypertension	415(43.3)	473(47.1)	2.709	0.100
Coronary heart disease	112(11.7)	133(13.2)	1.051	0.305
Diabetes	122(12.7)	100(10.0)	3.824	0.051
Hyperlipemia	27( 2.8)	34( 3.4)	0.514	0.474
Obesity	27( 2.8)	17( 1.7)	2.800	0.091

* t value, the other χ^2^ value.

**Table 2:** Comparison of health-related indices between management and control groups before and after 18 months

**Variables**	**Baseline**			**After intervention**		
	**Management group**	**Control group**	**P**	**Management group**	**Control group**	**P**
	**Mean (SD)**	**Mean (SD)**		**Mean (SD)**	**Mean (SD)**	
	**or n(%)**	**or n(%)**		**or n(%)**	**or n(%)**	
**subjective grade items**						
Health knowledge scores (0–29)	5.62(6.59)	5.35(6.38 )	0.28	22.02(7.89)	9.61(7.08)	0.00
Self-evaluated mental health status score (1–5)	3.51(1.01)	3.54(0.81)	0.08	3.54(0.94)	3.69(0.55)	0.00
Self-evaluated health status score (1–5)	3.42(0.83)	3.40(0.87)	0.74	3.67(0.88)	3.46(0.70)	0.00
Diet (0–18)	14.59(2.11)	14.67(2.43)	0.64	15.22(1.85)	14.87(1.74)	0.00
Physical activity duration (minutes per week)	119.32(257.9)	104.38(213.1)	0.03	269.45(266.2)	168.56(170.7)	0.00
Regular Monitoring blood pressure	415(43.4%)	394(39.2%)	0.06	820(85.7%)	629(62.6%)	0.00
Regular Monitoring blood glucose	209(21.8%)	200(19.9%)	0.29	690(72.1%)	176(17.5%)	0.00
**Objective measurement items**						
Body Mass Index (BMI)	24.13(3.28)	23.79(3.33)	0.01	23.51(4.26)	23.31(3.63)	0.28
Male BMI	23.60(2.99)	23.66(2.98)	0.55	23.31(4.23)	23.36(3.72)	0.85
Female BMI	24.64(3.46)	23.91(3.61)	0.00	23.69(4.29)	23.27(3.55)	0.09
Waist- to- hip ratio (WHR)	0.88(0.06)	0.89(0.06)	0.29	0.88(0.06)	0.96(0.10)	0.00
Male WHR	0.89(0.06)	0.90(0.06)	0.11	0.88(0.06)	0.96(0.09)	0.00
Female WHR	0.87(0.06)	0.87(0.06)	0.69	0.88(0.06)	0.96(0.11)	0.00
Systolic blood pressure (mmHg)	137.10(16.0) 128.45(14.85)		0.78	131.50(14.0)	126.80(9.55)	0.00
Diastolic blood pressure (mmHg)	84.61 ( 9.2)	81.11( 8.24)	0.00	80.85( 8.21)	80.53(5.87)	0.31
Fasting blood glucose (mmol/l)	7.11(2.44)	7.18(2.63)	0.85	5.64(1.30)	6.50(1.79)	0.00
	(n=112*)	(n=133*)		(n=112*)	(n=133*)	
Hyperlipemia patients triglyceride (mmol/l)	2.43(1.56)	2.21(1.89)	0.63	1.97(0.81)	1.96(0.72)	0.95
	(n=27#)	(n=34#)		(n=27#)	(n=34#)	
**Health Service Utilization**						
Number of outpatient clinic visits in the preceding 6 months	2.17(3.94)	2.14(3.48)	0.60	2.30(2.90)	2.70(2.37)	0.00
Number of admissions in the preceding 6 months	0.06(0.30)	0.06(0.40)	0.62	0.02(0.07)	0.03(0.21)	0.36

* Diabetes patients, # Hyperlipemia patients.

### Evaluation of the effect of health management

#### Comparison of health indices before and after intervention between the management and control groups

At the end of the 18-month study, three different categories of health-related indices were monitored: subjective grading items, objective measurement items, and health service utilization. Participants in the management group performed better (P<0.01) than those in the control group on all subjective grading items (health knowledge score, self-evaluated mental health status score, self-evaluated health status score, dietary score, physical activity duration per week and regular monitoring of blood pressure). Among the objective measured indices, improvements in the management group were observed in waist-to-hip ratio (WHR), systolic blood pressure and fasting blood sugar (P<0.01) compared with the control group. In terms of health service utilization, even though the number of outpatient clinic visits in the preceding 6 months was not significantly different between the two groups at baseline (P=0.60), the number in the management group was lower than that in the control group after intervention (P<0.01). However, the number of admissions to the hospital in the preceding 6 months showed no difference between the management and control groups (P=0.36) (Table 2).

### Multiple regression analysis of factors affecting on health index

Multiple regression analysis was performed to see the effect of gender (X1), age (X2), education level (X3), marriage status (X4), resident status (X5), chronic disease status (X6), self-evaluated psychological status at baseline (X7) and self-evaluated health conditions at baseline (X8) on the before–after differences in the management and the control group. For health knowledge, the score was significantly greater in association with the following factors: male, older, more educated, living with a family member, with chronic disease, and better self-evaluated psychological status at the baseline. Among these, education level and self-evaluated health conditions at baseline had the greatest influences on health knowledge score. For the body mass index of the elderly, the differences were significantly greater for males and for those with poor self-evaluated psychological status. For WHR, the difference was significantly greater for female gender, younger age, less education, with chronic disease, or poor self-evaluated psychological status; gender had the most effect on WHR. In terms of systolic pressure, the difference was significantly larger for female gender, younger age, less education, living with a family member, people with chronic disease, or poor self-evaluated psychological status; Chronic disease status had the strongest impact on systolic pressure. With regard to diastolic pressure, for those who were female, older or sick, the difference was significantly greater. For outpatient clinic visits, the difference was significantly greater in the group or with more education; chronic disease status(standardized partial regression coefficient was equal to 0.16) had more effect than education level (standardized partial regression coefficient was equal to −0.06) (Table 3).

**Table 3:** Multiple regression analysis of factors affecting health indices

	**Sex**	**Age**	**Education**	**Resident**	**Sick**	**BPS**	**BHS**
	X1	X2	X3	X5	X6	X7	X8
**Health knowledge scores**							
*β*	−0.06	0.06	−0.15	0.05	0.06	−0.15	
*P*	0.01	0.02	0.00	0.03	0.00	0.00	
**Body Mass Index**							
*β*	−0.05						0.06
*P*	0.03						0.01
**Waist-to- hip ratio**							
*β*	0.14	−0.07	0.04		0.07	0.05	
*P*	0.00	0.00	0.09		0.00	0.02	
**Systolic blood pressure (mmHg)**							
*β*	0.06	−0.05	0.06	−0.04	0.11	0.04	
*P*	0.01	0.02	0.02	0.09	0.00	0.07	
**Diastolic blood pressure**							
*β*	0.04	0.06			0.06		
*P*	0.09	0.01			0.01		
**Number of outpatient clinic visits**							
*β*			−0.06		0.16		
*P*			0.01		0.00		

**Note: *β*** is standardized partial regression coefficient; ***P*** is probability; **BPS** is self-evaluated psychological status; **BHS** is self-evaluated health conditions at the baseline.

## Discussion

Using a randomized controlled trial with community-based health management, we have demonstrated that health management improved several health indices and decreased outpatient utilization of health services during the 18-month study interval. However, the number of admissions to hospital did not change, most likely owing to the short length of the study. These findings are consistent with the results reported by Younmi [18]. Analyses showed that gender, age, education level, chronic disease status and self-evaluated psychological status are the main predictors of improvements in health indices (subjective and objective). This is compatible with the findings of Huang et al. [19]. More specifically, chronic disease status and self evaluated psychological status were positively affected by health management; several health indices showed improvement such as cognition, slow down in progression of the underlying disease, and confidence in and positive attitude towards a healthy life style. As a result, during implementation of health management, the measures for specific factors should be taken to increase the effect of health management. These results indicate that our community-based health management intervention was effective in improving the health of the elderly. Our community-based health management not only manages the health of the elderly by specially trained community health service center staff, managers and related researchers, but also educates them with some related knowledge and skills to enhance their health self-management ability.

Current studies on health management mainly focus on health care or service management and disease management [20-26]. Their main limitations are: narrow scope, microcosmic (medical institutions) point of view, and lack of systematic approach in experimental studies. Howe et al. indicated that health management should be expanded from disease management to population health management [27]. Some other studies have suggested that health management is effective for improving the health of the elderly [8,10], but their study results are not consistent [12,28]. Fletcher et al. showed that the differences in mortality and admission rates between the hospital management service and primary health service management groups were of no significance; however, the life quality of the elderly improved significantly with the management service in hospital compared with the primary health service management [28]. Analyzing the main social factors influencing the health of the elderly and evaluating the appraisal tool for health risk factors in health management, Iliffe et al. found that health management could improve the health of the elderly [29]. In our research, health management was not limited to specific diseases. Rather, it was expanded to the management of the elderly in general. Our intervention allowed for the development of tailor-made health improvement actions, including prevention and life style changes which proved to be effective.

There are a number of limitations in our trial. Although our research has demonstrated that health management can improve the health of the elderly in several categories (subjective grading, objective measurement, health service utilization and multiple regression analysis on factors affecting the health index differences), some long-term indices such as mortality have not yet been obtained and the number of admissions to hospital between the two groups was not significantly different. This most likely was due to the short duration of the study (only 18 months of health management intervention). In the future, extending the duration of the study could be helpful for further investigation of the long-term effects of health management on the health of the elderly.

## Conclusion

With a randomized controlled trial set in Nanjing, China, we have demonstrated that community-based health management can improve the health of the elderly. This is true both for scores of subjective grading (health knowledge score, self-evaluated psychological status, self-evaluated health status, dietary score, physical activity duration per week, and regular monitoring of blood pressure) and for objective health indices (WHR, systolic pressure, diastolic pressure, and fasting blood sugar), as well as for health service utilization (the number of outpatient clinic visits).

## Competing interests

The authors declare that they have no competing interests.

## Authors’ contributions

JC conducted the data analysis, drafted the manuscript and contributed to subsequent revisions. PL conceived the idea for the study, participated in study design, and contributed to the data analysis and to the drafting and revising of the manuscript. Other authors contributed to implementing the study, analyzing the data, and editing of the final manuscript. All authors read and approved the final manuscript.

## Pre-publication history

The pre-publication history for this paper can be accessed here:

http://www.biomedcentral.com/1472-6963/12/449/prepub
